# Lumen‐apposing metal stents provide early and late clinical benefits for the management of benign gastrointestinal strictures: Is there a role for definitive therapy?

**DOI:** 10.1002/deo2.70005

**Published:** 2024-09-01

**Authors:** Ethan Pollack, Dalton Norwood, Hector Caceres, Babusai Rapaka, Isaac E. Perry, Usman Barlass, Rachel Mitchell, Jessica McCreight, Shajan Peter, Ramzi Mulki, Ali Ahmed, Kondal Kyanam, Sergio A. Sánchez‐Luna

**Affiliations:** ^1^ Department of Internal Medicine The University of Alabama at Birmingham Heersink School of Medicine Birmingham Alabama USA; ^2^ Department of Internal Medicine UAB Minority Health and Health Equity Research Center The University of Alabama at Birmingham Heersink School of Medicine Birmingham Alabama USA; ^3^ Department of Internal Medicine Division of Gastroenterology & Hepatology Basil I. Hirschowitz Endoscopic Center of Excellence The University of Alabama at Birmingham Heersink School of Medicine Birmingham Alabama USA

**Keywords:** gastrointestinal disorders, gastrointestinal endoscopes, gastrointestinal tract, stents, strictures

## Abstract

**Objectives:**

This study aimed to characterize the clinical outcomes, safety, and efficacy of lumen‐apposing metal stents (LAMS) in treating benign gastrointestinal strictures.

**Methods:**

A single‐center retrospective review of all patients who underwent LAMS placement for benign strictures from June 2017 to July 2023. Primary outcomes were technical success, early clinical success, late clinical success (LCS), and sustained post‐LAMS clinical success (SPLCS). Secondary outcomes included stent dwell time, stenosis changes, adverse events, reintervention rates, and symptomatology evaluation.

**Results:**

Thirty‐five patients underwent placement of 42 LAMS (74% female, mean age: 54.2 ± 11.7 years). Anastomotic strictures accounted for 64% of cases (*N* = 27, 45% at the gastrojejunal anastomosis). The median STD was 91.0 days (interquartile range [IQR]: 55.0–132.0). Technical success was obtained in all cases. Early clinical successand LCS were achieved in 80% of cases overall. SPLCS was achieved in 45% (*n* = 15) of cases. The overall reintervention rate was 63%, with a median time to reintervention being 50.5 days (IQR: 24–105). adverse events occurred in 28% (*n* = 12) overall, with a 24% migration rate (*n* = 10). Follow‐up was completed in 83% of cases with a median duration of 629 days (range: 192.0–1297.0). Overall symptom improvement occurred in 79% (*n* = 27) during indwelling LAMS versus 58% and 56% at 30‐ and 60‐days post‐removal, respectively.

**Conclusions:**

LAMS for benign gastrointestinal strictures are associated with high technical and early clinical success/LCS rates, positive quality‐of‐life metrics, and a tolerable adverse event rate. Overall, recurrence of symptoms and high reintervention rates post‐LAMS removal reinforce the difficulty in managing benign gastrointestinal strictures but also argue for LAMS as a definitive therapy in select cases.

## INTRODUCTION

Benign gastrointestinal (GI) strictures lead to significant morbidity – including dysphagia, severe malnutrition, abdominal pain, and reduced quality of life. These strictures are classified into anastomotic (post‐surgical) versus non‐anastomotic (peptic ulcer disease, radiation therapy, caustic injury). Endoscopic balloon dilation (EBD) is the standard, first‐line treatment for re‐establishing luminal patency. Refractory strictures are challenging to manage and may require non‐surgical management options, including intralesional steroid injections, needle‐knife stricturotomy, or self‐expandable metal stents (SEMS).^1^, ^2^, ^3^, ^4^, ^5^ These modalities have demonstrated limitations and sub‐optimal outcomes.^6^, ^7^ EBD necessitates frequent esophagogastroduodenoscopies (EGDs) and carries a risk of recurrence, bleeding, and perforation.^7^ SEMS were a novel alternative to refractory strictures because of their ease of deployment and ability to provide gradual, continuous dilation. Unfortunately, treatment efficacy has been limited by migration, dwell times, tissue ingrowth, and symptom recurrence post‐removal.^8^, ^9^, ^10^, ^11^ Surgical management options exist but carry risks and limitations, including post‐operative morbidity and patient candidacy.^1^, ^12^

In recent years, luminal‐apposing metal stents (LAMS) have emerged as a novel approach to treating refractory, intrinsic GI strictures.^13^, ^14^, ^15^, ^16^, ^17^ Initially designed for gallbladder and peri‐pancreatic fluid drainage, LAMS’ dumbbell‐shaped design may improve migration rates, patient tolerability, dwell times, and overall efficacy in managing benign, intrinsic GI strictures.^18^, ^19^ However, single‐center studies remain limited in assessing the overall efficacy, tolerability, safety, and patient‐specific quality‐of‐life metrics following LAMS placement.^20^

This study aimed to address LAMS's safety, efficacy, feasibility, and tolerability in managing benign, intrinsic GI strictures. We sought to characterize clinical features that better predict early clinical success (ECS), late clinical success (LCS), and sustained post‐LAMS clinical success (SPLCS) following LAMS placement. Additionally, we hope to provide insight into who may benefit from LAMS as a definitive therapy.

## METHODS

### Data collection and patients

Under IRB approval at The University of Alabama at Birmingham (UAB), we performed a retrospective review of a prospectively collected database of all patients who underwent LAMS placement for benign strictures from 06/2017 to 07/2023. Electronic medical records (EMRs) were used to obtain patient information, including baseline characteristics, clinical/surgical history, and endoscopic procedures. Procedural information, including stenosis dimensions, LAMS characteristics, dilation, reintervention, STD, and AEs, was obtained from procedural documentation/imaging and follow‐up notes in the EMR. A standardized questionnaire was utilized for patient telephone follow‐ups (Supporting Information Questionnaire). LAMS placement was exclusively performed by the five experienced interventional endoscopists at our center.

### Patients

Patients with stents other than LAMS, malignant strictures, or no follow‐up endoscopy were excluded. Patients who underwent multiple LAMS placements during separate procedures or underwent LAMS reintervention were included. Endoscopy, imaging, and histologic biopsies were used to diagnose benign intrinsic GI strictures. Patients were informed of and consented to the off‐label utilization of LAMS to treat these strictures.

### Definitions

In all cases, patients had failed EBD prior to consideration for LAMS placement. Stricture location and etiology were determined based on surgical history, stricture characteristics, and anatomic location before and during endoscopy. Stenosis diameter and length were measured endoscopically prior to stent placement. These measurements were re‐reviewed by a separate, expert endoscopist to ensure accuracy before formal analysis. Contrast‐assisted stricturogram was used to measure stenosis length when applicable.

Technical success was defined as the endoscopic confirmation of successful stent placement during the procedure with appropriate LAMS deployment. ECS was defined as symptom resolution 30 days after stent placement, including patients with indwelling stents on day 30 and patients who had elective removal prior to day 30. LCS was defined as symptom resolution greater than 30 days and up to 60 days after stent placement, inclusive of patients with indwelling stents and those who had stent removal before day 60. SPLCS was defined as symptom resolution at 30 days and up to 60 days after stent removal, irrespective of stent dwell time (SDT).

Patient symptoms were characterized via a standardized questionnaire and follow‐up protocol developed by our institution. All AEs were recorded and graded according to the American Society for Gastrointestinal Endoscopy (ASGE) lexicon.^21^ Migration was identified by follow‐up endoscopy. Migration was defined as a change in the luminal location of LAMS outside of the initial stricture site and detachment from the stricture site. The removal of LAMS was based on a predetermined interval in some cases, but patient‐physician discussions regarding symptom resolution, burden, or AEs were utilized for optimal endoscopic follow‐up in most cases. The decision for re‐intervention in any form was determined by the endoscopist in conjunction with patient discussions.

**FIGURE 1:**
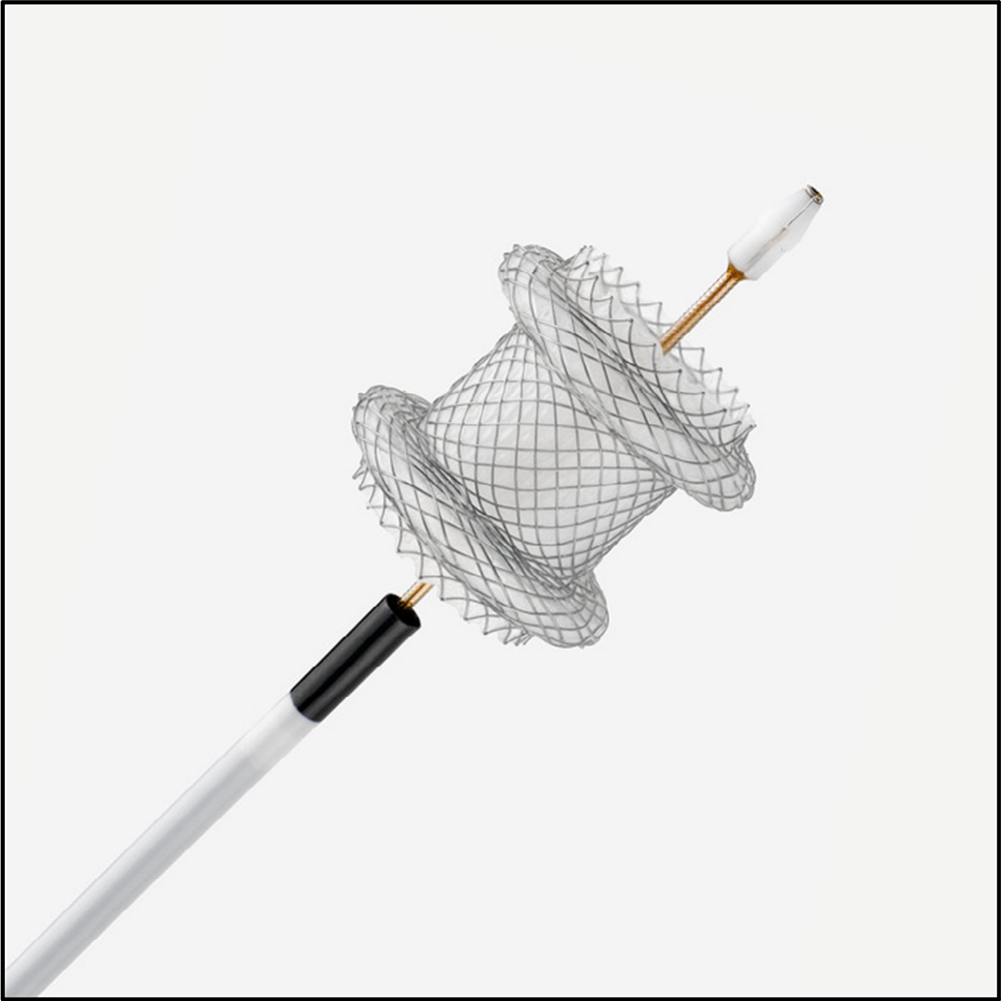
Displays the structure of the AXIOS lumen‐apposing metal stent system (Boston Scientific Inc.), which is a biflanged dumbbell‐shaped fully‐covered stent made of Nitinol wire and covered with silicone. This lumen‐apposing metal stent and introducer system were used in all cases.

**FIGURE 2:**
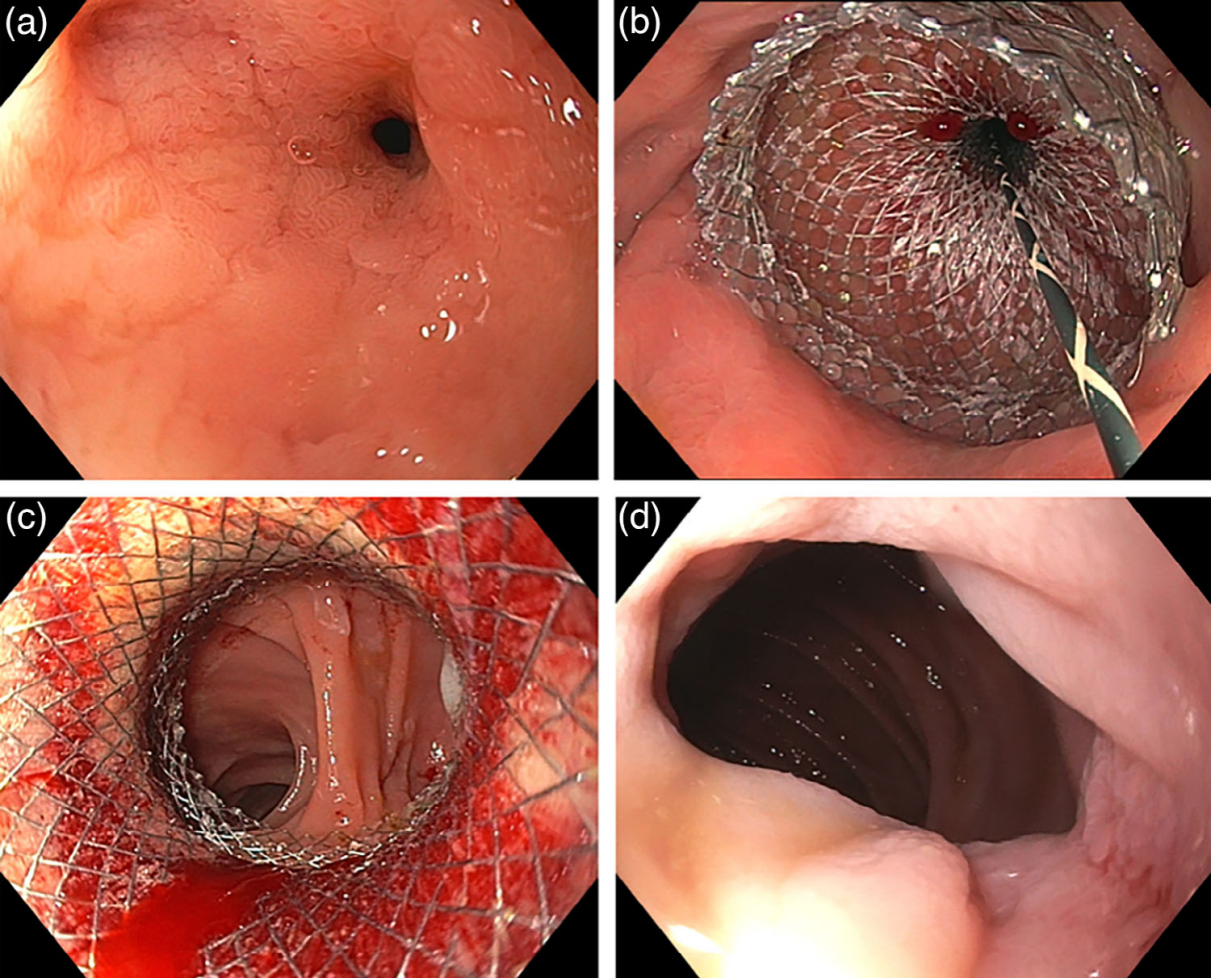
(a) High‐grade gastrojejunal anastomotic stricture. (b) Placement of a 20 × 10 mm non‐cautery enhanced lumen‐apposing metal stent over a 0.035‐inch guide wire with (c) subsequent through the scope balloon dilation up to 18 mm of the saddle of the lumen‐apposing metal stent and visualization of downstream jejunum. (d) Status post removal of lumen‐apposing metal stent 3 months after index episode showing a resolved gastrojejunal anastomotic stricture.

The AXIOS LAMS system (Boston Scientific Inc.) was utilized for all cases (Figure 1). The study included stent lengths of 10 or 15 mm and three different stent diameters (10/15/20 mm). The endoscopist chose the stent size based on the stricture characteristics. Using a dual‐channel therapeutic endoscope, the LAMS was loaded and deployed following a standard over‐the‐wire technique under direct endoscopic and fluoroscopic guidance when necessary. LAMS was deployed such that both flanges were closely approximated to the rim of the stricture and secured by the lumen‐apposing properties inherent to the stent itself (Figure 2). In a marginal portion of cases,^3^ LAMS was anchored with an endoscopic suturing system (OverStitch; Boston Scientific Inc.) or with an over‐the‐scope clip (OTSC; OVESCO Endoscopy AG) placement. The decision to dilate was based on the expertise and clinical judgment of the advanced endoscopist. A through‐the‐scope (TTS) balloon dilator was used for incremental dilation up to 19mm in these cases. All strictures were traversed following stent placement to evaluate for bleeding, perforation, mucosal changes, and adequate approximation of stent with mucosal tissue. Patients were counseled on smoking cessation and avoidance of non‐steroidal anti‐inflammatories prior to and following stent placement.

### Statistical analysis

Continuous variables were expressed as mean ± standard deviation or median with interquartile range (IQR) for skewed data. Categorical variables were presented as frequencies and percentages. Chi‐square or Fisher's exact tests were used to explore differences for categorical variables or t‐tests/non‐parametric tests for continuous variables. OR with 95% confidence intervals were calculated to evaluate associations, particularly regarding reintervention and factors influencing clinical success.

## RESULTS

### General characteristics

Thirty‐five patients underwent placement of 42 LAMS for benign GI strictures. Anastomotic strictures accounted for 64% (*n* = 27) of cases versus 36% (*n* = 15) non‐anastomotic. Females accounted for 74% of the total cohort, which did not differ between groups (*p* = 0.43). The mean age for anastomotic strictures was 50.9 ± 10.8 years versus non‐anastomotic cases 60.1 ± 11.1 (*p* = 0.012). Patient distribution across age categories (18–45, 45–54, 55–64, and >65) was not statistically significant between the non‐anastomotic and anastomotic groups (*p* = 0.14; Table 1).

**TABLE 1:** General characteristics of the population differentiated by etiology (non‐anastomotic vs. anastomotic).

	Total	Non‐anastomotic	Anastomotic	*p*‐value
	*N* = 42	*N* = 15	*N* = 27	
Gender				0.43
Female	31 (74%)	10 (67%)	21 (78%)	
Male	11 (26%)	5 (33%)	6 (22%)	
Age	54.2 (11.7)	60.1 (11.1)	50.9 (10.8)	0.012
Age category				0.14
18–45	10 (24%)	1 (7%)	9 (33%)	
45–54	13 (31%)	4 (27%)	9 (33%)	
55–64	14 (33%)	7 (47%)	7 (26%)	
>65	5 (12%)	3 (20%)	2 (7%)	
Anatomic region				<0.001
GJA and EJA	22 (52%)	0 (0%)	22 (81%)	
Esophagus	3 (7%)	2 (13%)	1 (4%)	
Gastric and duodenal	14 (33%)	13 (87%)	1 (4%)	
Colorectal	3 (7%)	0 (0%)	3 (11%)	
Specific location				<0.001
GJA	19 (45%)	0 (0%)	19 (70%)	
Colorectal	3 (7%)	0 (0%)	3 (11%)	
duodenal bulb	4 (10%)	3 (20%)	1 (4%)	
Jejunal	2 (5%)	2 (13%)	0 (0%)	
Pyloric	8 (19%)	8 (53%)	0 (0%)	
gastroesophageal junction	1 (2%)	0 (0%)	1 (4%)	
Esophageal	3 (7%)	2 (13%)	1 (4%)	
EJA	2 (5%)	0 (0%)	2 (7%)	
Stenosis diameter (mm)	3.0 (3.0‐5.0)	3.0 (3.0‐4.0)	4.0 (3.0‐6.0)	0.096
<5 mm	30 (71%)	14 (93%)	16 (59%)	0.019
≥5 mm	12 (29%)	1 (7%)	11 (41%)	
Stenosis length (mm)	5.0 (3.0‐8.0)	5.0 (3.0‐5.0)	5.0 (5.0‐10.0)	0.07
>5 mm	14 (33%)	3 (20%)	11 (41%)	0.17
≤5 mm	28 (67%)	12 (80%)	16 (59%)	
Pre‐LAMS symptoms				
Dysphagia	24 (71%)	8 (62%)	16 (76%)	
Semi‐solids	7 (32%)	2 (29%)	5 (33%)	
Liquids	7 (32%)	2 (29%)	5 (33%)	
Total dysphagia	8 (36%)	3 (43%)	5 (33%)	
Vomiting	28 (82%)	11 (85%)	17 (81%)	
abdominal pain	23 (68%)	7 (54%)	16 (76%)	
abdominal distension	14 (41%)	6 (46%)	8 (38%)	
Constipation	21 (62%)	7 (54%)	14 (67%)	
Thin stool caliber	14 (41%)	6 (46%)	8 (38%)	

The most common stricture site was at the gastro‐jejunal (GJA) or esophago‐jejunal anastomosis (EJA; 52%), followed by gastric and duodenal (33%), esophageal (7%), and colorectal (7%). GJA and EJA strictures accounted for 81% (*n* = 22) of cases in the anastomotic group (*n* = 22), while gastric and duodenal strictures accounted for 87% (*n* = 13) of non‐anastomotic cases. The median stenosis diameter was 3.0 mm (IQR: 3.0–5.0 mm) for the entire cohort. In the non‐anastomotic group, 93% of patients had stenosis <5 mm versus 59% in the anastomotic group (*p* = 0.019). The median stenosis length was 5.0 mm overall (IQR: 3.0–8.0 mm), which did not differ statistically between groups (*p* = 0.07; Table 1).

### Procedural information and follow‐up

60% (*n* = 25) of cases required a 20 × 10 mm LAMS. The LAMS was dilated in 38% (*n* = 16) of cases (Table 2). Regarding anchoring, endoscopic suturing was used in two cases, and an OTSC was used in one case.

**TABLE 2:** Procedure and telephone follow‐up outcomes.

	Total	Non‐Anastomotic	Anastomotic	*p*‐value
	*N* = 42	*N* = 15	*N* = 27	
**Procedure follow‐up**				
LAMS size				0.74
10 × 10	1 (2%)	0 (0%)	1 (4%)	
15 × 10	12 (29%)	5 (33%)	7 (26%)	
20 × 10	25 (60%)	8 (53%)	17 (63%)	
15 × 15	4 (10%)	2 (13%)	2 (7%)	
LAMS dilated				0.64
No	26 (62%)	10 (67%)	16 (59%)	
Yes	16 (38%)	5 (33%)	11 (41%)	
Technical success	42 (100%)	15 (100%)	27 (100%)	
Post‐LAMS stenosis diameter (mm)	12.0 (10.0–15.0)	12.0 (11.0–15.0)	12.0 (10.0–15.0)	0.61
Change in stenosis diameter (mm)	8.0 (6.0–11.0)	9.0 (8.0–11.0)	7.0 (6.0–11.0)	0.34
>5 mm	29 (76%)	12 (80%)	17 (74%)	0.67
Stent dwell time (days)	91.0 (55.0–132.0)	64.5 (34.0–100.0)	100.0 (73.0–132.0)	0.1
>60 days	27 (69%)	8 (57%)	19 (76%)	0.22
>90 days	20 (51%)	4 (29%)	16 (64%)	0.034
Migration	10 (24%)	3 (20%)	7 (26%)	0.67
Adverse events				0.15
None	40 (95%)	13 (87%)	27 (100%)	
Pneumonitis	1 (2%)	1 (7%)	0 (0%)	
Retained stent	1 (2%)	1 (7%)	0 (0%)	
Change in weight (lbs)	0.2 (‐2.0–9.5)	0.0 (‐4.8–7.0)	3.0 (0.0–10.6)	0.19
No change*	10 (28%)	4 (29%)	6 (27%)	0.2
Loss >1 lb	10 (28%)	6 (43%)	4 (18%)	
Gained >1 lb	16 (44%)	4 (29%)	12 (55%)	
Days to followed‐up	629.0 (192.0–1297.0)	868.0 (232.0–1304.0)	360.5 (173.0–852.0)	0.2
Vital Status				
Alive	35 (95%)	11 (92%)	24 (96%)	
Dead	2 (5%)	1 (8%)	1 (4%)	
Early clinical success	28 (80%)	9 (69%)	19 (86%)	0.22
Late clinical success	28 (80%)	9 (69%)	19 (86%)	0.22
Post‐LAMS success	15 (45%)	6 (46%)	9 (45%)	0.95
Reintervention	24 (63%)	5 (33%)	19 (83%)	0.002
Type of reintervention				
LAMS	10 (42%)	2 (40%)	8 (42%)	
Dilation	11 (46%)	2 (40%)	9 (47%)	
Dilation and stricturoplasty	3 (12%)	1 (20%)	2 (11%)	
Days to reintervention	49.0 (20.0–105.0)	24.0 (20.0–38.0)	57.5 (26.0–105.0)	0.25

The median SDT was 91.0 days (IQR: 55.0–132.0). The longest SDT was 598 days. LAMS was tolerated well overall and remained in place for at least 30 days in 38 cases (90.4%). Furthermore, 69% (*N* = 27) of cases had an SDT of greater than 60 days, and 51% (*N* = 20) remained in place for greater than 90 days (Table 2). Technical success was achieved in 100% (*n* = 42) of cases. Post‐LAMS stenosis diameter increased by 8.0 mm (IQR: 6.0–11.0). The median post‐LAMS stenosis diameter was 12 mm (IQR: 10.0‐15.0). Both ECS and LCS were achieved in 80% (*n* = 28) of cases. When stratified based on stricture type, 69% of non‐anastomotic strictures and 86% of anastomotic strictures achieved both ECS and LCS (*p* = 0.22). SPLCS was achieved in 45% (*n* = 15) of patients (Table 2).

AEs were documented in a total of 12 cases (28%). Migration occurred in 10 cases (24%). The stenosis diameter before intervention for individuals who did not have migration was 3.3 versus 4.5 mm in those who migrated (*p* = 0.043). Although not statistically significant, in patients with a pre‐LAMS stenosis diameter greater than 5 mm, migration occurred in 40% of cases versus 15% of cases where pre‐LAMS stenosis diameter was less than 5 mm (*p* = 0.059). Excluding migration, two stent‐related AEs were noted. The first was considered a Grade II AE. The patient was discharged after one day in the hospital for aspiration pneumonitis following EGD. The second was considered a Grade III AE requiring surgical intervention. Due to refractory symptoms and difficulty with stent removal in a Billroth‐I‐related stricture, the patient subsequently underwent the creation of a Roux‐en‐Y gastrojejunostomy.

**FIGURE 3:**
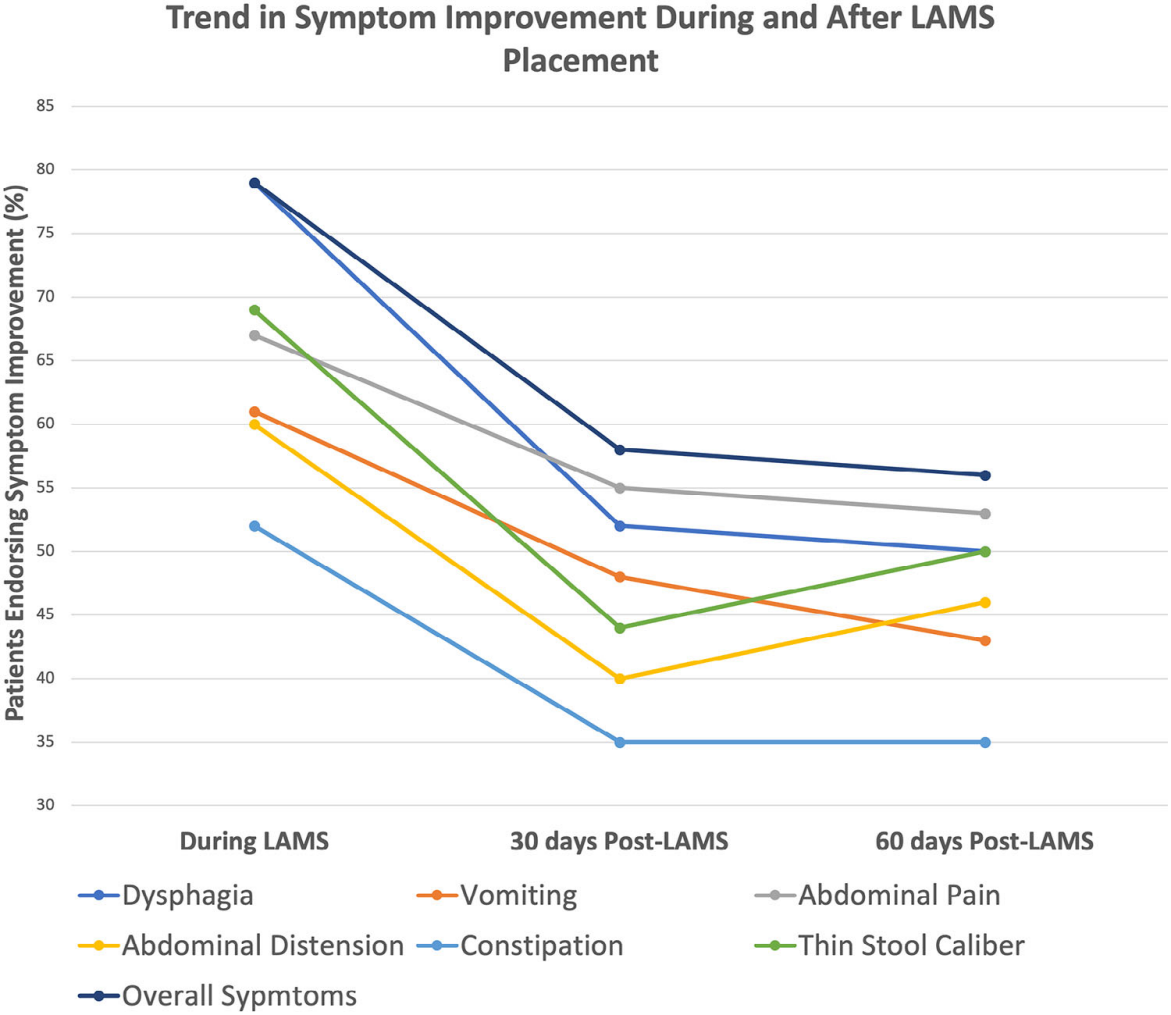
Depicts symptom behaviors during lumen‐apposing metal stent (LAMS) as well as 30‐ and 60‐days post‐LAMS removal. Depicts patient‐driven symptom improvement during LAMS as well as 30‐ and 60‐days post‐LAMS removal

A standardized follow‐up questionnaire was completed by 83.3% (*n* = 35) of patients. The median number of days from stent placement to questionnaire follow‐up was 629 days (192–1297 days). Table 1 displays patient‐specific symptoms prior to LAMS placement. While LAMS was in place, dysphagia improved in 75% (*n* = 18) of patients, which was maintained at 30 days and 60 days after LAMS removal in 52% (*n* = 11) and 50% (*n* = 9), respectively. Overall, symptoms improved during LAMS placement in 79% (*n* = 27) of cases. This improvement was maintained post‐removal at 30 and 60 days in 58% (*n* = 18) and 56% (*n* = 15) of cases, respectively (Table 2 and Figure 3).

Table 3 presents the frequency and odds ratio [OR] of factors associated with ECS and SPLCS. A longer SDT (>60 days) showed a significant association with ECS (OR = 16.80, 95% confidence interval [CI]: 1.53–184.92, *p* = 0.021). Additionally, weight gain of more than 1 pound exhibited a significant trend toward ECS (OR = 16.32, 95% CI: 1.11–152.35, *p* = 0.041). Regarding SPLCS, migration showed a trend toward significance (OR = 0.19, 95% CI: 0.03–1.11, *p* = 0.050).

**TABLE 3:** Table of frequency and odds ratio of factors associated with early clinical success and sustained post‐lumen‐apposing metal stent clinical success.

Early clinical success	No	Yes	Odds ratio	95% CI		*p*‐value
Anastomotic	3 (43%)	9 (32%)	2.81	0.52	15.32	0.231
Stenosis diameter (mm), mean (SD)	4.14 (3.75)	3.75 (1.62)	0.87	0.53	1.42	0.571
Stenosis diameter (>5 mm)	2 (29%)	8 (29%)	1.00	0.16	6.25	0.980
Change in stenosis diameter (mm), mean (SD)	5.30 (4.30)	8.58 (3.88)	1.22	0.98	1.53	0.076
Change in stenosis diameter (>5 mm)	4 (57%)	21 (81%)	3.15	0.53	18.80	0.208
Stent dwell time (days), mean (SD)	38 (18)	128 (115)	1.08	1.01	1.15	**0.020**
Stent dwell time (>60 days)	1 (20%)	21 (81%)	16.80	1.53	184.92	**0.021**
Migration	4 (57%)	6 (21%)	0.20	0.04	1.17	0.075
Change in weight						
Loss weight	4 (57%)	4 (16%)	Reference			
No change	2 (29%)	8 (32%)	4.24	0.50	31.98	0.191
Gained >1 lb	1 (14%)	13 (52%)	16.32	1.11	152.35	**0.041**

Sustained Post‐LAMS clinical success	No	Yes				
Anastomotic	11 (61%)	9 (60%)	0.95	0.23	3.88	0.948
Stenosis diameter (mm), mean (SD)	3.9 (1.7)	3.5 (1.5)	0.83	0.53	1.29	0.405
Stenosis diameter (>5 mm)	2 (28%)	3 (20%)	0.65	0.13	3.33	0.605
Change in stenosis diameter (mm), mean (SD)	6.9 (4.3)	9 (3.8)	1.15	0.95	1.39	0.164
Change in stenosis diameter (>5 mm)	12 (67%)	13 (87%)	3.25	0.55	19.32	0.195
Stent dwell time (days), mean (SD)	121 (142)	98 (64)	1.00	0.99	1.00	0.567
Stent dwell time (>60 days)	10 (62%)	10 (77%)	2.00	0.39	10.31	0.407
Migration	8 (44%)	2 (13%)	0.19	0.03	1.11	**0.050**
Change in weight						
Loss weight	6 (33%)	2 (14%)	Reference			
No change	5 (28%)	5 (36%)	3.09	0.39	22.71	0.287
Gained >1 lb	7 (39%)	7 (50%)	2.92	0.44	20.31	0.260

Reintervention occurred in 63% (*n* = 24) of cases following LAMS removal, which was more likely in anastomotic strictures (OR = 9.5, 95% CI: 2.07–43.50, *p* = 0.004). The median time to reintervention was 50.5 days (IQR: 24–105). Post‐LAMS stenosis diameter was 13.5 ± 3.7 mm in those not requiring reintervention compared to 10.9 ± 3.4 mm in those who required reintervention (*p* = 0.04). Follow‐up for LAMS‐specific reinterventions was available in five cases at the time of publication. This cohort's overall migration rate was 20% (*n* = 1). ESC and LCS were achieved in 60% of those cases (*n* = 3), while SPLCS was achieved in one case (20%). No additional AEs were reported.

## DISCUSSION

This study demonstrates a safe and effective alternative to conventional therapies for benign GI strictures. Our technical (100%) and ECS (80%) were comparable to the previous literature utilizing LAMS.^13^, ^15^, ^16^, ^20^, ^22^ Furthermore, our results were maintained during the 30‐ to 60‐day period in all patients who initially achieved ECS. With a few exceptions, patients achieving LCS still had the LAMS in situ at the 60‐day mark. This study demonstrates that LAMS was durable for extended dwell times with maintained clinical improvement throughout this period.^23^

SPLCS was limited (45%). However, in patients who did not experience migration of LAMS, SPLCS was more likely (57% no migration vs. 20% migration; *p* = 0.05). Reintervention was also common (63%), with a LAMS‐specific reintervention of 24% reflecting the difficulty in treating refractory strictures post‐stent removal. This is in comparison to SEMS, which has demonstrated a post‐stent clinical success rate of 0%–40%, and EBD, which requires a median of three sessions to achieve adequate dilation with symptom improvement in only 66.5% of cases.^9^, ^23^, ^24^, ^25^ Taken together, LAMS was tolerated well and demonstrated improved and sustained efficacy post‐removal compared to EBD and SEMS.

Although a cost‐effective analysis was not included, current literature demonstrates that LAMS becomes economically equivalent after 3.5 EBDs for non‐anastomotic strictures and two EBDs for anastomotic strictures.^26^ At our institution, the cost ratio of LAMS placement and removal compared to EBD is 2.16, lower than the 5.7 reported previously.^26^ Although reintervention rates would likely affect cost‐effectiveness, our breakeven point is likely less than the published 3.5 dilations. Further cost analyses should consider reintervention rates and SDT to better assess LAMS's overall economic impact.

Our study's median SDT of 91.0 days (IQR: 55.0–132.0) was comparable to prior studies utilizing LAMS, where average SDT ranged from 60 to 120 days.^15^, ^16^, ^20^, ^22^ LAMS demonstrated continued tolerability with dwell times exceeding previous SEMS (∼30–90 days).^23^, ^27^, ^28^ However, in our study, dwell time was not a predictor of SPLCS or the need for reintervention. Given LAMS were tolerated for extended dwell times, up to 598 days, engaging in patient‐centered discussions regarding initial symptom response to determine optimal SDT is beneficial. Although an initial LAMS‐free trial is likely warranted since 45% of patients experienced SPLCS, some patients with refractory post‐removal symptoms may benefit from LAMS replacement as a definitive therapy. In these cases, patients would require continued endoscopic and radiographic monitoring with consideration of stent exchange to avoid complications such as tissue overgrowth or stent degradation/delamination.

**FIGURE 4:**
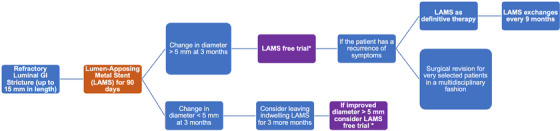
Proposed treatment algorithm for managing refractory, benign gastrointestinal (GI) strictures with lumen‐apposing metal stent (LAMS). *Proposed treatment algorithm for the management of refractory, benign GI strictures with the utilization of LAMS*.

Although not statistically significant based on a standard confidence interval of 95%, ECS and LCS were associated with a change in stenosis diameter of 8.6 versus 5.3 mm in those who did not (*p* = 0.064). Furthermore, post‐LAMS stenosis diameter also differed between those requiring reintervention (10.9 ± 3.4 mm) versus those who did not require reintervention (13.5 ± 3.7 mm; *p* = 0.04). An adequate stricture dilation during LAMS removal appears pivotal in determining clinical success. However, with large stricture diameters, migration and clinical failure risk also increases. Due to the aforementioned factors and our experience, we propose the following algorithm for treating benign GI stricture in patients’ refractory to traditional endoscopic therapies described in Figure 4.

Migration rates (24%) were consistent with published data.^13^, ^15^, ^17^, ^20^, ^22^ This is in comparison to SEMS, which demonstrates a previously reported migration rate of 30%–40% and up to 53% without suturing.^24^, ^27^, ^28^, ^29^ There was a significant difference between initial stenosis diameter in patients who did not have stent migration compared to those who experienced migration (3.3 mm without migration vs. 4.5 mm with migration). Providers should consider initial stenosis diameter when determining migration risks, particularly in patients with an initial stenosis diameter greater than 5 millimeters. The utilization of LAMS as a re‐intervention strategy may be limited by increased migration rates since luminal diameter at reintervention is likely increased from initial stent placement. Our LAMS‐specific reintervention data was limited to 5 cases. In this cohort, only one patient (20%) had evidence of migration at follow‐up and no additional adverse events or procedural limitations were noted. Future prospective studies are needed to determine long‐term stent tolerability, durability, cost‐effectiveness, and migration rates following LAMS re‐intervention and LAMS as definitive therapy.

We acknowledge the inherent limitations of the retrospective study design conducted at a single tertiary care center. An additional risk of bias stems from the small sample size, female predominance, heterogeneity of stent locations, and lack of universal measurement guidelines for stricture diameter. Interventions and reinterventions at other sites are possible. However, extensive follow‐up documentation was available and reviewed in the EMR to limit this confounder. Additionally, follow‐up duration, stent removal, and indications for reintervention were driven by symptom burden, stricture response, and patient‐physician discussions rather than an established timeframe or protocol. This may have led to bias in SDTs, AEs, and reinterventions. Along these lines, the decision for stent dilation was not standardized but determined intraprocedural by the endoscopist based on stent apposition to the luminal wall and stricture diameter post‐stent placement. We limited the inherent bias of telephone questionnaires by minimizing the number of investigators completing telephone surveys^3^ and utilizing a standardized questionnaire/grading scale (Supplemental 2).

LAMS is a safe and effective management option for benign GI strictures with longer dwell times and favorable migration rates compared to previous SEMS. LAMS is both tolerated well and improves GI symptoms that directly affect quality of life. Unfortunately, SPLCS is limited. Patients tend to experience the return of GI symptoms within the first 1–2 months following stent removal. Based on our study and institutional experience, we propose a LAMS‐specific reintervention algorithm to assist practitioners in treating benign GI strictures refractory to initial LAMS intervention. In some cases, LAMS as a definitive therapy should be considered for those who have failed previous stent‐free periods. Given the clinical success in retrospective studies, further randomized controlled trials are warranted to compare LAMS against more conventional endoscopic therapies.

## CONFLICT OF INTEREST STATEMENT

Dr. Sánchez‐Luna is the recipient of the 2021 American Society for Gastrointestinal Endoscopy (ASGE) Endoscopic Training Award by the ASGE and Fujifilm. This was not relevant to this study. The rest of the authors declare no conflict of interest.

## ETHICS STATEMENT

The study was approved by the University of Alabama at Birmingham (UAB) Institutional Review Board (IRB‐300010961), meaning all authors and the research protocol complied with UAB's ethical guidelines. Informed consent was obtained in all cases. Study registration was not applicable to this study.

## Supporting information


**SUPPLEMENTAL QUESTIONNAIRE**: Post‐LAMS (AXIOS) follow‐up telephone instrument.
**TABLE S1** Symptoms during LAMS placement, 30‐ and 60‐days post‐LAMS removal.

## References

[1] de Wijkerslooth LR , Vleggaar FP , Siersema PD . Endoscopic management of difficult or recurrent esophageal strictures. Am J Gastroenterol 2011; 106: 2080–2091; quiz 92. 22008891 10.1038/ajg.2011.348

[2] Ramage JI, Jr. , Rumalla A , Baron TH *et al.* A prospective, randomized, double‐blind, placebo‐controlled trial of endoscopic steroid injection therapy for recalcitrant esophageal peptic strictures. Am J Gastroenterol 2005; 100: 2419–2425. 16279894 10.1111/j.1572-0241.2005.00331.x

[3] Ferguson DD . Evaluation and management of benign esophageal strictures. Dis Esophagus 2005; 18: 359–364. 16336604 10.1111/j.1442-2050.2005.00516.x

[4] Kochhar R , Poornachandra KS . Intralesional steroid injection therapy in the management of resistant gastrointestinal strictures. World J Gastrointest Endosc 2010; 2: 61–68. 21160692 10.4253/wjge.v2.i2.61 PMC2999060

[5] Kochhar R , Kochhar S . Endoscopic balloon dilation for benign gastric outlet obstruction in adults. World J Gastrointest Endosc 2010; 2: 29–35. 21160676 10.4253/wjge.v2.i1.29 PMC2998862

[6] Holm AN , de la Mora Levy JG , Gostout CJ , Topazian MD , Baron TH . Self‐expanding plastic stents in treatment of benign esophageal conditions. Gastrointest Endosc 2008; 67: 20–25. 17945227 10.1016/j.gie.2007.04.031

[7] Boregowda U , Goyal H , Mann R *et al*. Endoscopic management of benign recalcitrant esophageal strictures. Ann Gastroenterol 2021; 34: 287–299. 33948052 10.20524/aog.2021.0585 PMC8079876

[8] Gangloff A , Lecleire S , Di Fiore A *et al*. Fully versus partially covered self‐expandable metal stents in benign esophageal strictures. Dis Esophagus 2015; 28: 678–683. 25168061 10.1111/dote.12260

[9] Hirdes MM , Siersema PD , Vleggaar FP . A new fully covered metal stent for the treatment of benign and malignant dysphagia: A prospective follow‐up study. Gastrointest Endosc 2012; 75: 712–718. 22284093 10.1016/j.gie.2011.11.036

[10] Ham YH , Kim GH . Plastic and biodegradable stents for complex and refractory benign esophageal strictures. Clin Endosc 2014; 47: 295–300. 25133114 10.5946/ce.2014.47.4.295 PMC4130882

[11] van Halsema EE , van Hooft JE . Clinical outcomes of self‐expandable stent placement for benign esophageal diseases: A pooled analysis of the literature. World J Gastrointest Endosc 2015; 7: 135–153. 25685270 10.4253/wjge.v7.i2.135 PMC4325310

[12] Hua S , Lye EC . Impact of gastric and bowel surgery on gastrointestinal drug delivery. Drug Deliv Transl Res 2023; 13: 37–53. 35585472 10.1007/s13346-022-01179-6 PMC9726802

[13] Larson B , Adler DG . Lumen‐apposing metal stents for gastrointestinal luminal strictures: Current use and future directions. Ann Gastroenterol 2019; 32: 141–146. 30837786 10.20524/aog.2018.0337 PMC6394263

[14] Siersema PD , de Wijkerslooth LR . Dilation of refractory benign esophageal strictures. Gastrointest Endosc 2009; 70: 1000–1012. 19879408 10.1016/j.gie.2009.07.004

[15] Tan S , Zhong C , Huang S *et al*. Clinical outcomes of lumen‐apposing metal stent in the management of benign gastrointestinal strictures: A systematic review and meta‐analysis. Scand J Gastroenterol 2019; 54: 811–821. 31290352 10.1080/00365521.2019.1638447

[16] Santos‐Fernandez J , Paiji C , Shakhatreh M *et al*. Lumen‐apposing metal stents for benign gastrointestinal tract strictures: An international multicenter experience. World J Gastrointest Endosc 2017; 9: 571–578. 29290912 10.4253/wjge.v9.i12.571 PMC5740102

[17] Jain D , Patel U , Ali S , Sharma A , Shah M , Singhal S . Efficacy and safety of lumen‐apposing metal stent for benign gastrointestinal stricture. Ann Gastroenterol 2018; 31: 425–438. 29991887 10.20524/aog.2018.0272 PMC6033762

[18] Leung Ki EL , Napoleon B . EUS‐specific stents: Available designs and probable lacunae. Endosc Ultrasound 2019; 8: S17–S27. 31897375 10.4103/eus.eus_50_19 PMC6896438

[19] Hindryckx P , Degroote H . Lumen‐apposing metal stents for approved and off‐label indications: A single‐centre experience. Surg Endosc 2021; 35: 6013–6020. 33051767 10.1007/s00464-020-08090-6

[20] Mahmoud T , Beran A , Bazerbachi F *et al*. Lumen‐apposing metal stents for the treatment of benign gastrointestinal tract strictures: A single‐center experience and proposed treatment algorithm. Surg Endosc 2023; 37: 2133–2142. 36316581 10.1007/s00464-022-09715-8

[21] Nass KJ , Zwager LW , van der Vlugt M *et al*. Novel classification for adverse events in GI endoscopy: The AGREE classification. Gastrointest Endosc 2022; 95: 1078–1085 e8. 34890695 10.1016/j.gie.2021.11.038

[22] Yang D , Nieto JM , Siddiqui A *et al*. Lumen‐apposing covered self‐expandable metal stents for short benign gastrointestinal strictures: A multicenter study. Endoscopy 2017; 49: 327–333. 28114688 10.1055/s-0042-122779

[23] Fuccio L , Hassan C , Frazzoni L , Miglio R , Repici A . Clinical outcomes following stent placement in refractory benign esophageal stricture: A systematic review and meta‐analysis. Endoscopy 2016; 48: 141–148. 26528754 10.1055/s-0034-1393331

[24] Fujii LL , Bonin EA , Baron TH , Gostout CJ , Wong Kee Song LM . Utility of an endoscopic suturing system for prevention of covered luminal stent migration in the upper GI tract. Gastrointest Endosc 2013; 78: 787–793. 23871095 10.1016/j.gie.2013.06.014

[25] Pereira‐Lima JC , Ramires RP , Zamin I, Jr. , Cassal AP , Marroni CA , Mattos AA . Endoscopic dilation of benign esophageal strictures: Report on 1043 procedures. Am J Gastroenterol 1999; 94: 1497–1501. 10364013 10.1111/j.1572-0241.1999.01061.x

[26] Hallac A , Srikureja W , Liu E , Dhumal P , Thatte A , Puri N . Economical effect of lumen apposing metal stents for treating benign foregut strictures. World J Gastrointest Endosc 2018; 10: 294–300. 30364856 10.4253/wjge.v10.i10.294 PMC6198308

[27] Bakken JC , Wong Kee Song LM , de Groen PC , Baron TH . Use of a fully covered self‐expandable metal stent for the treatment of benign esophageal diseases. Gastrointest Endosc 2010; 72: 712–720. 20883848 10.1016/j.gie.2010.06.028

[28] Eloubeidi MA , Lopes TL . Novel removable internally fully covered self‐expanding metal esophageal stent: Feasibility, technique of removal, and tissue response in humans. Am J Gastroenterol 2009; 104: 1374–1381. 19491851 10.1038/ajg.2009.133

[29] Dan DT , Gannavarapu B , Lee JG , Chang K , Muthusamy VR . Removable esophageal stents have poor efficacy for the treatment of refractory benign esophageal strictures (RBES). Dis Esophagus 2014; 27: 511–517. 23121426 10.1111/j.1442-2050.2012.01432.x

