# Supplementary material for: Lumen‐apposing metal stents provide early and late clinical benefits for the management of benign gastrointestinal strictures: Is there a role for definitive therapy?
Source: DEN Open. 2024 Sep 1;5(1):e70005. doi: 10.1002/deo2.70005 (PMC11366066; doi:10.1002/deo2.70005)
Supplement: Supplementary file 1 — SUPPLEMENTAL QUESTIONNAIRE: Post‐LAMS (AXIOS) follow‐up telephone instrument. TABLE S1 Symptoms during LAMS placement, 30‐ and 60‐days post‐LAMS removal. [file DEO2-5-e70005-s001.docx]

|  | During LAMS | 30 days Post-LAMS | 60 days Post-LAMS | p-value |
| --- | --- | --- | --- | --- |
| Dysphagia (n=24) |  |  |  | 0.083 |
| No Change | 5 (21%) | 6 (29%) | 4 (22%) |  |
| Improved | 19 (79%) | 11 (52%) | 9 (50%) |  |
| Worsened | 0 ( 0%) | 4 (19%) | 5 (28%) |  |
| Vomiting (n=28) |  |  |  | 0.3 |
| No Change | 10 (36%) | 8 (32%) | 7 (33%) |  |
| Improved | 17 (61%) | 12 (48%) | 9 (43%) |  |
| Worsened | 1 ( 4%) | 5 (20%) | 5 (24%) |  |
| Abdominal Pain (n=23) |  |  |  | 0.78 |
| No Change | 6 (29%) | 7 (35%) | 6 (32%) |  |
| Improved | 14 (67%) | 11 (55%) | 10 (53%) |  |
| Worsened | 1 ( 5%) | 2 (10%) | 3 (16%) |  |
| Abdominal Distension (n=14) |  |  |  | 0.65 |
| No Change | 5 (33%) | 5 (33%) | 4 (31%) |  |
| Improved | 9 (60%) | 6 (40%) | 6 (46%) |  |
| Worsened | 1 ( 7%) | 4 (27%) | 3 (23%) |  |
| Constipation (n=21) |  |  |  | 0.2 |
| No Change | 10 (48%) | 8 (40%) | 9 (45%) |  |
| Improved | 11 (52%) | 7 (35%) | 7 (35%) |  |
| Worsened | 0 ( 0%) | 5 (25%) | 4 (20%) |  |
| Thin stool caliber (n=14) |  |  |  | 0.66 |
| No Change | 4 (25%) | 6 (38%) | 5 (36%) |  |
| Improved | 11 (69%) | 7 (44%) | 7 (50%) |  |
| Worsened | 1 ( 6%) | 3 (19%) | 2 (14%) |  |
| Overall Symptom (n=34) |  |  |  | 0.12 |
| No Change | 5 (15%) | 7 (23%) | 4 (15%) |  |
| Improved | 27 (79%) | 18 (58%) | 15 (56%) |  |
| Worsened | 2 ( 6%) | 6 (19%) | 8 (30%) |  |

**Supplemental Table 1. *Symptoms during LAMS placement, 30- and 60-days post-LAMS removal.***

**Supplemental Questionnaire: Post-LAMS (AXIOS) Follow Up Call Instrument.**

Post-LAMS (AXIOS) Follow up call Instrument.

PI: Sergio Sanchez-Luna,MD

Personal identifiers:

| ID (Excel): |  |
| --- | --- |
| MRN (Excel): |  |
| Procedure Date (Excel): |  |
| Name (Excel): |  |
| Phone Number (Excel): |  |

Hi, Good (morning/afternoon)! This is (Dr. XXX name) from the Gastroenterology Division in the University of Birmingham at Alabama, are you Mr/Ms(patient Name)?

| Responded Call | 0- No  1- Yes |
| --- | --- |
| Call Date (Follow-up date) | ___ / ___/ ____ (MM/DD/YYYY) |
| follow-up days | Leave Blank) |
| Source of Information | 0- Patient  1- Proxy |

If yes, continue with call, if not; is Mr/Ms. (patient name) alive?

| Vital Status | 0-Alive  1- Dead |
| --- | --- |

If yes, is there any way to get in contact with him right now or is there any time I can give him a call just to follow-up on him/her?

If not alive, express empathetic condolences and ask if to the best of his/her knowledge can answer some questions of the patient following his treatment.

We are calling you because you attended had a stent placed in our endoscopy unit on (Procedure date) and we are attempting to evaluate the impact of the procedure in you and other individuals, do you recall this procedure?

No, I understand, I would like to thank you very much for your valuable time, is there a better time or date I can call you? Yes, perfect I will schedule a call for that time, No; thank you very much, hope you have a great rest of your day if you change your mind you can call or text this phone number (XXX)XXX-XXXX.

| Patient/proxy accepted follow-up? | 0- No 1- Yes |
| --- | --- |

Perfect, I will be very briefly we just want to know some symptoms prior to the procedure, while you had the stent placed and 30 and 60 days after the removal, we understand that you might not recall very specific this time frames,

I will try to help you remember and answer to the best of your knowledge.

If patient died:

| Died + Not removed | 0- No  1- Yes |
| --- | --- |

Prior to the procedure on (month and year of procedure) did you have:

- **Dysphagia** (Do you have difficulty swallowing food? Or does food get stuck after you swallow?)
  - **If yes:** Related to the prior symptom which of the following best describes your difficulty eating: 0- normal diet, 0- some solids, 2- semi solids, 3- liquids, 4- anything.
- **Abdominal pain**
- **Abdominal Distension**
- **Constipation**
- **This stool caliber**

|  | Pre-LAMS |
| --- | --- |
| Symptoms | 1- Dysphagia  2- Vomiting  3- Abdominal Pain  4- Abdominal Distension  5- Constipation  6- Thin stool caliber |
| if dysphagia | 0, normal diet  1, ability to swallow some solids;   2, semi-solids;   3, liquids;   4, total dysphagia.  5, Not Applicable |

Now I want to know how your symptoms changed.

When you had the procedure done while the stent was placed and not yet removed how would you say that (read column in bold), now 30 days after the removal (read the same column, mark the 30 day column), now 60 days after the removal (read 60 day removal column).

|  | During LAMS | 30 days after removal | 60 days after removal |
| --- | --- | --- | --- |
| if dysphagia: **would you say your dysphagia:** | 0- No change  1- Improved  2- Worsened | 0- No change  1- Improved  2- Worsened  3- N/A | 0- No change  1- Improved  2- Worsened  3- N/A |
| if Vomiting: **would you say your vomiting:** | 0- No change  1- Improved  2- Worsened | 0- No change  1- Improved  2- Worsened  3- N/A | 0- No change  1- Improved  2- Worsened  3- N/A |
| if abdominal pain: **would you say your abdominal pain:** | 0- No change  1- Improved  2- Worsened | 0- No change  1- Improved  2- Worsened  3- N/A | 0- No change  1- Improved  2- Worsened  3- N/A |
| if abdominal distension: **would you say your abdominal distension:** | 0- No change  1- Improved  2- Worsened | 0- No change  1- Improved  2- Worsened  3- N/A | 0- No change  1- Improved  2- Worsened  3- N/A |
| if constipation: **would you say your constipation:** | 0- No change  1- Improved  2- Worsened | 0- No change  1- Improved  2- Worsened  3- N/A | 0- No change  1- Improved  2- Worsened  3- N/A |
| if thin stool caliber: **would you say your stool diameter:** | 0- No change  1- Improved  2- Worsened | 0- No change  1- Improved  2- Worsened  3- N/A | 0- No change  1- Improved  2- Worsened  3- N/A |
| Overall, would you say your symptoms reduced? | 0- No change  1- Improved  2- Worsened | 0- No change  1- Improved  2- Worsened  3- N/A | 0- No change  1- Improved  2- Worsened  3- N/A |

Finally, are you interested in learning about research studies at UAB? These studies may be surveys, studies to assess the effectiveness of a drug, or studies that do not involve drugs but test the effectiveness of other programs such as diet, physical activity or strength building programs, support programs, and similar programs. You can always refuse participation if we contact you and you are not interested.

0- No
 1- Yes

That will be all the questions I have for you today, thank you very much for your time and remember that for any questions regarding your treatment or symptoms you can log-in your patient portal or call to make an appointment.
